# Uranium-Doped Zinc, Copper, and Nickel Oxides for Enhanced Catalytic Conversion of Furfural to Furfuryl Alcohol: A Relativistic DFT Study

**DOI:** 10.3390/molecules27186094

**Published:** 2022-09-18

**Authors:** Shuang Li, Yu-Chang Hou, Yuan-Ru Guo, Qing-Jiang Pan

**Affiliations:** 1 Key Laboratory of Functional Inorganic Material Chemistry (Ministry of Education), School of Chemistry and Materials Science, Heilongjiang University, Harbin 150080, China; 2 Engineering Research Center of Advanced Wooden Materials, Ministry of Education, College of Material Science and Engineering, Northeast Forestry University, Harbin 150040, China

**Keywords:** transition metal oxide catalyst, uranium doping, furfural and furfuryl alcohol, thermodynamic reaction pathway, relativistic DFT

## Abstract

Transition metal oxides (TMOs) and actinide ones (AnOs) have been widely applied in catalytic reactions due to their excellent physicochemical properties. However, the reaction pathway and mechanism, especially involving TM–An heterometallic centers, remain underexplored. In this respect, relativistic density functional theory (DFT) was used to examine uranium-doped zinc, copper, and nickel oxides for their catalytic activity toward the conversion of furfural to furfuryl alcohol. A comparison was made with their undoped TMOs. It was found that the three TMOs were capable of catalyzing the reaction, where the free energies of adsorption, hydrogenation, and desorption fell between −33.93 and 45.00 kJ/mol. The uranium doping extremely strengthened the adsorption of CuO-U and NiO-U toward furfural, making hydrogenation or desorption much harder. Intriguingly, ZnO-U showed the best catalytic performance among all six catalyst candidates, as its three reaction energies were very small (−10.54–8.12 kJ/mol). The reaction process and mechanism were further addressed in terms of the geometrical, bonding, charge, and electronic properties.

## 1. Introduction

With the continuous exploitation and consumption of fossil resources, environmental problems such as global warming have increasingly intensified [1,2,3,4]. The issues of energy crisis and scarcity of fossil resources have attracted more and more attention. Pursuing green and sustainable development is one of the most fundamental solutions. Biomass materials are organic carbon materials that are readily available in nature, and their abundant, clean, and renewable advantages [5] provide a form of solution. Lignocellulose is an economical and noncompetitive biomass material in the food chain. It is rich in hemicellulose (20–35%) and can be used as a raw material for furfural (FAL) [6,7]. In addition, there are four types of pentose-rich biomass for the preparation of furfural, i.e., xylan, mannan, xyloglucan, and β-glucan [8]. Furfural is an important biomass platform molecule [9,10]. As a component molecule in the biochemical industry, it acts as a bridge between biomass raw materials and downstream products. It is the product with the highest output in biorefinery with the exception of bioethanol [11]. It can be used as raw material to produce furfuryl alcohol (FOL), 2-methylfuran, 2-methyltetrahydrofuran, tetrahydrofurfuryl alcohol, cyclopentanone, etc. [12,13]. Among these products, furfuryl alcohol has the largest production demand. More than 50% of the annual output of furfural is used to produce furfuryl alcohol [14,15]. Furfuryl alcohol is a precursor to prepare a series of high value-added chemical products, including lysine, vitamins C, lubricants, fibers, resins, rubber, dispersants, and rocket fuel [16,17].

Two schemes have been realized for the hydrogenation conversion of furfural to furfuryl alcohol, namely, direct hydrogenation with hydrogen as the hydrogen source and catalytic transfer hydrogenation with hydrogen-containing substances as the hydrogen source [18,19]. The former is divided into two technical routes of liquid-phase and gas-phase hydrogenation [17,20]. Furfural liquid-phase hydrogenation technology has a history of about 90 years and is an earlier solution to realize industrialization. Its shortcomings are that it requires higher hydrogen pressure, uses toxic solvents, and has high operating costs for batch reactors. The application of gas-phase hydrogenation technology solves the above problems and realizes the continuous reaction under mild conditions [20]. The catalytic transfer hydrogenation technology of furfural uses green and cheap alcohol or organic acid as the hydrogen source and is a promising carbonyl reduction hydrogenation technology [21]. The difficult storage and transport of dihydrogen can be avoided [22]. Regardless of the hydrogenation method, the corresponding catalyst is the key to efficient conversion. There are more noble metal catalysts and transition metal nano-catalysts in the direct hydrogenation method [23,24]. The former is difficult to industrialize due to its high cost [25]. As a manner of fact, the current commercially used catalyst for the production of furfuryl alcohol from furfural is a Cr-containing Cu-based catalyst [12]. It is believed that the reductive hydrogenation ability of Cu-based catalysts is more suitable, while the reducibility of Ni-based catalysts is too strong, which will lead to the decomposition of products after hydrogenation [26]. Alloying methods and selecting suitable catalyst supports are common methods for people to explore new catalysts [27,28,29,30,31]. In these studies, Ni, Cu, Zn, and their oxides often appear as catalyst components. Comparative tests in some studies showed poor catalytic performance of zinc and copper oxides, but the BET surface area of the catalyst samples suggested that this performance difference seems to be due to the insufficient exposure of its surface active sites with the excessively large particle size [19,20]. However, studies on the catalytic performance of transition metal oxides based on consistent particle size scale and microstructure have not been reported.

On the basis of experimental findings, transition metal oxides (TMOs) of Ni, Cu, and Zn were constructed in terms of finite cluster model as catalysts for the catalytic hydrogenation of furfural. Their intrinsic catalytic performance and mechanism were investigated using density functional theory. Recently, it was reported that the introduction of electron-rich 4f-block metals into the catalyst is an effective method to improve the catalytic performance [32]. However, the research applying 4f-block metals remains very rare. To this end, the modification of catalysts by U doping was computationally studied. This study is expected to provide the guidance for the design of highly efficient catalysts for the hydrogenation of furfural to furfuryl alcohol.

## 2. Computational Details

**Scheme 1:**
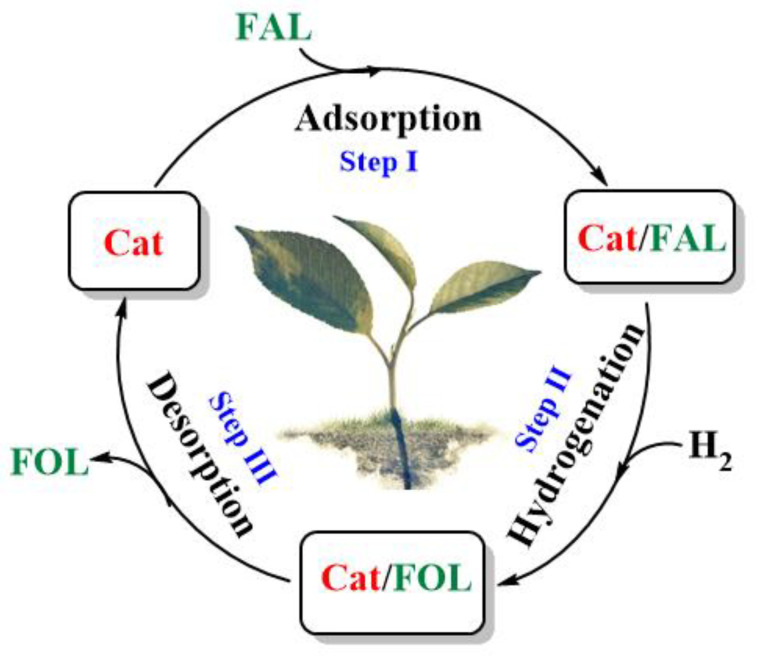
Reaction pathway of the catalytic conversion from furfural to furfuryl alcohol.

The catalytic hydrogenation of furfural in this study includes three steps as seen in Scheme 1. In Step I, the active center of the catalyst adsorbs the furfural, and a catalyst–furfural complex is formed, denoted as Cat/FAL. Step II is that Cat/FAL reacts with dihydrogen, and the hydrogenation generates a catalyst and furfuryl alcohol complex (marked as Cat/FOL). Step III is that the newly functionalized furfuryl alcohol desorbs from the catalyst, thus closing the catalytic cycle.

**Figure 1:**
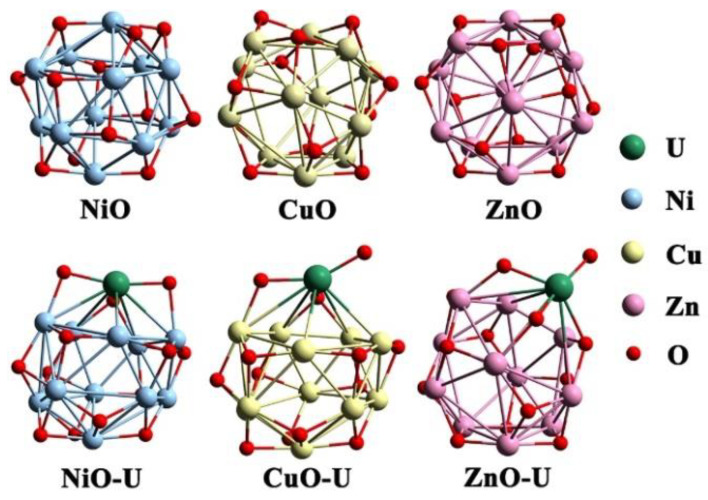
Optimized structures of transition metal oxide catalysts and uranium-doped ones.

**Figure 2:**
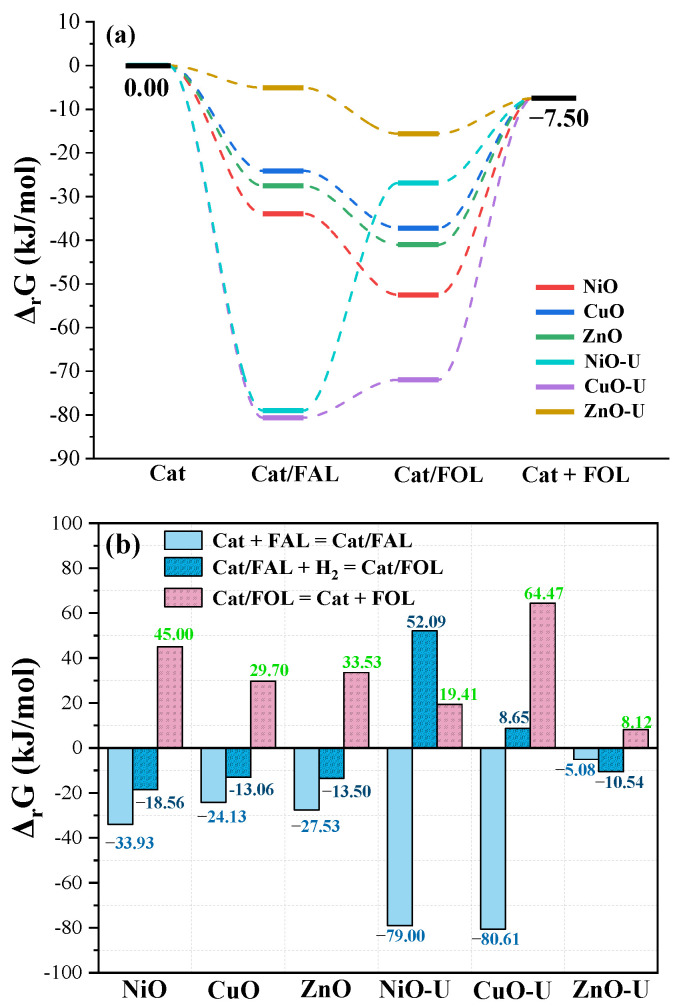
Gibbs free energy (kJ/mol) for all catalysts along the reaction pathway (**a**), together with free energies of adsorption, hydrogenation, and desorption reactions (**b**).

In this work, we used a finite cluster model to simulate transition metal oxides (TMOs). Twelve units, (TMO)_12_ (TM = Zn, Cu, and Ni), were employed as reported in previous work [33,34,35]. They were denoted as ZnO, CuO, and NiO, respectively. On the basis of these, uranium doping was introduced, yielding (TMO)_11_UO_2_ (marked as TMO-U). The optimized structures of the abovementioned six catalysts are presented in Figure 1. Cat/FAL and Cat/FOL complexes of each catalyst would have several energetically stable isomers. All optimized structures are illustrated in Figures S1–S6 of the Supplementary Materials, marked as Cat/FAL-n and Cat/FOL-n. The most stable structure of each complex is given in Figure 2. In these cases, the number (n = 1) is omitted for convenience.

All the structures including catalysts, reagents, intermediates, and products were fully optimized, and their frequencies were calculated using the quantum chemistry software Priroda 6.0 [36]. The calculations did not adopt symmetry constraints. All structures presented in the paper had no imaginary frequencies. The thermodynamic data (298.15 K), Mulliken charge, and Mayer bond order were obtainable. The free energy, for instance, was attained by the sum of the electronic total energy and the free energy correction. In the calculations, we used relativistic Hamiltonian, GGA PBE functional, and all-electron correlated bipolar Gaussian basis sets. The self-consistent field convergence criterion was set to 10^−6^ au.

**Figure 3:**
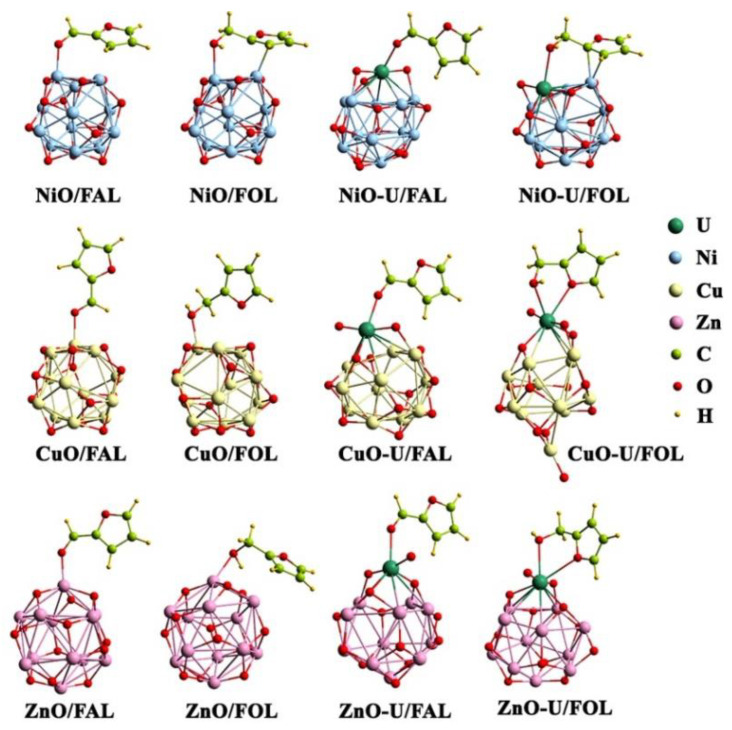
Optimized structures for intermediates Cat/FAL and Cat/FOL, where the most stable isomer for each intermediate is presented.

The electronic structures of the 12 most stable structures in Figure 3 were explored using ADF 2012 [37] software. The single-point calculations were performed without further re-optimizations. The electronic structures were also obtained. Herein, the GGA PBE functional was using in the consideration of the D3BJ dispersion correction [38]. The scalar relativistic effect of ZORA was applied, along with the Slater-type TZP, basis sets, and the small frozen core. In previous work [34,39,40], the level of theory used herein successfully predicted structures of metal oxide clusters, calculated reaction energetics, and explained interfacial properties. Thus, it was deemed reliable and reasonable for the current study.

## 3. Results and Discussion

### 3.1. Thermodynamic Reactions

In this study, the activity of the catalyst was evaluated according to the thermodynamic energetics. In Figure 2a, the pathway in terms of Gibbs free energies (Δ_r_G) is depicted to show the whole catalytic pathway of each catalyst. The Δ_r_G values of each step (adsorption, hydrogenation, and desorption) are illustrated in Figure 2b.

We embark on a discussion of the three TMO catalysts. One can see that, in the first step (adsorption) of the catalytic cycle, the calculated Δ_r_G values of forming Cat/FAL were all negative, indicating that these reactions are spontaneous. The three TMO catalysts had good adsorption towards furfural. Of them, NiO was the most favored with the adsorption energy Δ_r_G of −33.93 kJ/mol (Figure 2b). The furfural adsorption energy of CuO was the largest, being −24.13 kJ/mol, while the adsorption energy of ZnO was a little more negative than that of CuO, which was −27.53 kJ/mol. Notably, a more negative Δ_r_G value denotes a stronger adsorption interaction.

In the second step (hydrogenation) of the three TMOs, furfuryl alcohol was generated on the surface of the catalyst. Calculations show that all the Gibbs reaction energies were still negative. The generation of Cat/FOL further reduced the energy of the system, and the reaction of this step proceeded spontaneously. As for Δ_r_G ordering of the three reaction systems, the hydrogenation step was consistent with the preceding step. The Gibbs reaction energies of CuO and ZnO in the hydrogenation step were −13.06 and −13.50 kJ/mol, respectively. The reaction energy of NiO was the smallest and most favorable (−18.56 kJ/mol).

The Δ_r_G value of the system after the two-step reaction directly affected the third step (desorption). For the catalytic reactions of these three TMO catalysts, desorption was a nonspontaneous process that required external energy to drive the catalytic cycle. For CuO, its desorption energy was the smallest, 29.70 kJ/mol, where the release of newly functionalized furfuryl alcohol occurred the most favorably. The second was the reaction pathway involving ZnO with a desorption energy of 33.53 kJ/mol. When NiO was used, the desorption of the product furfuryl alcohol was the most difficult. A desorption energy of 45.00 kJ/mol was required to drive the occurrence of the desorption reaction.

According to above comparisons, it can be found that when the three TMOs are used as a catalyst, the first two steps in the catalytic cycle can proceed spontaneously. They are thermodynamically driven reactions, which reflect the catalytic hydrogenation activity toward furfural. Although their desorption energies are positive, the product can still be desorbed from the catalyst surface and avoid the phenomenon of catalyst poisoning due to not so large values. This ensures the activity and stability of the TMO catalysts and realizes long-life recyclable use. It can be concluded that NiO, CuO, and ZnO possess intrinsic catalytic performance. From an energetic point of view, CuO was ranked as having the best activity, followed by ZnO and NiO.

One can note that, despite bearing catalytic ability, the above TMO catalysts still had to take a large-energy uphill step (29.70–45.00 kJ/mol). Then, we resorted to introducing an additional 5f-block metal center, i.e., uranium doping. Not surprisingly, the reaction pathways of the uranium-doped catalysts changed greatly. Among the six catalysts, the catalyst ZnO-U showed the best catalytic ability when inspecting the thermodynamic reaction pathways. Its catalytic conversion performance was greatly improved with respect to ZnO. Spontaneous adsorption (−5.08 kJ/mol Δ_r_G) and hydrogenation (−10.54 kJ/mol) were achieved for ZnO-U, with a desorption energy of only 8.12 kJ/mol, which is expected to give high-efficiency desorption, save energy, accelerate the catalytic cycle efficiency, and reduce the possibility of catalyst poisoning.

Uranium doping had different impacts on the other two catalysts (NiO-U and CuO-U). Firstly, their adsorption toward furfural was very strong with free energies of −79.00 and −80.61 kJ/mol, respectively. The subsequent hydrogenation and desorption steps were both nonspontaneous reactions. NiO-U was difficult to hydrogenate and easy to desorb, with a hydrogenation energy of 52.09 kJ/mol and the desorption energy 19.41 kJ/mol. In contrast, CuO-U was easy to hydrogenate and difficult to desorb, with a hydrogenation energy of 8.65 kJ/mol and a desorption energy of 64.47 kJ/mol. The performance gradual law of the three TMO catalysts, the differential modification of the catalysts after uranium doping, and the performance boost of ZnO-U are all interesting. Subsequently, the geometry, charges, and electronic structures are discussed to explain the thermodynamic possibility of the catalytic conversion.

In addition, a kinetic process was considered. The transition states of the furfural adsorption step of ZnO and ZnO-U were searched. As shown in Figure S10, the activation free energy was calculated to be 33.39 kJ/mol for ZnO. It was significantly reduced to 0.48 kJ/mol due to the uranium doping. The reaction kinetics confirms the improved catalytic performance by the U doping.

### 3.2. Geometric Structures

In Figure 3, we present the optimized structures of adsorption and hydrogenation intermediates of the six catalysts. It can be seen intuitively that the TMO catalysts displayed a spherical and well-proportioned structure after adsorption and hydrogenation. After uranium doping, the spherical structure of TMO-U was distorted to a certain extent; it was stretched and deformed along the direction of the spherical diameter passing through the U atom. This may have been caused by the large size of uranium or its repulsion with the neighboring metal atoms. NiO had the best structural compatibility with U atoms, and the resultant NiO-U structure showed less axial stretching. ZnO also showed good compatibility with doping structures, while CuO was seriously distorted after doping. The structural distortion of CuO-U/FOL was obvious. Structural distortion may have introduced additional open sites and caused further destruction of catalyst morphology during the reaction process. Consequently, the formed intermediates would have been too stable in energy to release the furfuryl alcohol (64.47 kJ/mol energy barrier). Thus, this resulted in the loss of catalytic performance of CuO-U and reduced the recyclability of the catalyst with respect to CuO.

The bond lengths between furfural/furfuryl alcohol and catalyst are given in Table 1. All these intermediate complexes contained metal–oxygen bonds (TM–O and U–O), which were mainly responsible for the stability of the system and determined the interaction of catalysts toward organic molecules to the greatest extent. There is competition between carbonyl oxygen (marked as O1) and furan oxygen (marked as O2) in the catalytic hydrogenation of furfural. In order to generate furfuryl alcohol, we expect that the carbonyl oxygen interacts with the catalyst, which facilitates the subsequent dihydrogen addition. From the structure obtained by our optimizations, the furfural was bound to the catalyst through its carbonyl oxygen without exception, implying the good catalytic selectivity of these six catalysts. By comparing the optimized bond length of M–O with the sum of atomic covalent radius and van der Waals radii (Table S4), it was found that the bond length before U doping was intermediate between the two, becoming less than the covalent radius after doping. The U doping somewhat strengthened the chemical bond strength.

### 3.3. Bond Orders and Charges

Stronger bonding may result in more stability of the system, and the corresponding reaction Gibbs free energy may become more negative. Bond order can used to characterize bond strength. With it, the relationship between the structure and the system energy can be analyzed. Before uranium doping, in the furfural adsorption step, TM–O1 had the strongest bonding in NiO/FAL with a bond order of 0.49, while the bond orders of CuO/FAL and ZnO/FAL were 0.41 and 0.36, respectively. There was also TM–C bond with a bond order of 0.10 in NiO/FAL, while a hydrogen bond order of 0.09 was found in ZnO/FAL. There was no additional bonding between CuO and FAL. Therefore, in Step I (adsorption), NiO/FAL had the most negative adsorption energy, followed by ZnO/FAL, and CuO/FAL had the most positive adsorption energy (Figure 2 and Table S2). The adsorption energies were relatively close, because the O···H hydrogen bond was weak and only provided moderate stability for ZnO/FAL compared with CuO/FAL.

In Step II (hydrogenation), the TM–O1 bond was weakened due to the reaction, while the other bonds were strengthened. The Ni–O bond order in NiO/FOL was only 0.27, and its lower energy resulted from the significantly enhanced Ni–C bonds; two bonds were formed with bond orders of 0.33 and 0.37. The bond order of TM–O in CuO/FOL was 0.31, whereas, in ZnO/FOL, it was 0.34, and the bond order of O···H in ZnO/FOL was 0.18. Therefore, the Gibbs reaction energy sequence was NiO/FOL < ZnO/FOL < CuO/FOL.

Upon doping by uranium, in the adsorption step, the U–O1 bond appeared between TMO-U and FAL. Its bond order in NiO-U/FAL, CuO-U/FAL, and ZnO-U/FAL was 0.52, 0.55, and 0.43, respectively. Accordingly, CuO-U/FAL had the lowest adsorption energy, while ZnO-U/FAL had the most positive adsorption energy, with NiO-U/FOL intermediate between them. In the hydrogenation step, the above analysis failed, and the Gibbs reaction energy order of Cat/FOL could not be correctly judged on the basis of the bond order. This may have been related to the structural distortion of the catalyst caused by U doping. At this time, compared with the bonding between the catalyst and furfuryl alcohol, the contribution of structural distortion to the energy of the system dominated.

**Figure 4:**
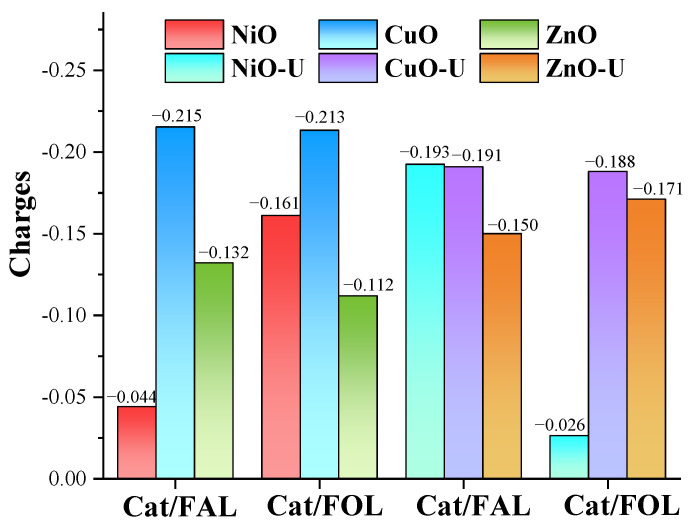
Mulliken charges of the catalyst fragment in intermediates Cat/FAL and Cat/FOL.

In Figure 4, the Mulliken charges of the catalyst part in Cat/FAL and Cat/FOL are depicted. The charge distribution on the catalyst moiety was negative, resulting from the electron transfer from furfural/furfuryl alcohol to the catalyst. The electron transfer contributed to the interaction between Cat and FAL/FOL. Among them, the charge value of the copper-based catalyst was larger in absolute value, ranging from −0.215 to −0.188, and hydrogenation had almost no effect on the charge. The charge of the zinc-based catalyst changed slightly, becoming more negative due to uranium doping: −0.132 (ZnO/FAL) vs. −0.112 (ZnO/FOL) and −0.150 (ZnO-U/FAL) vs. −0.171 (ZnO-U/FOL).

The charge value of nickel-based catalysts was very sensitive to hydrogenation and doping. The difference before and after hydrogenation was up to 0.167, while that before and after doping was up to 0.149. The sensitivity of nickel-based catalysts indicates that hydrogenation and doping significantly changed its electrostatic interaction strength with furfuryl alcohol/furfural, thereby significantly affecting the binding stability. For **NiO** complexes, the charge after hydrogenation of the adsorbed furfural increased significantly, from −0.044 to −0.161. Obviously, the electrostatic attraction of **NiO** with furfuryl alcohol was stronger than that with furfural. This led to a decrease in the energy **of NiO/FOL**, consistent with our calculated thermodynamics. Using **NiO-U** catalyst, the charge on the catalyst part after hydrogenation was significantly reduced, from −0.193 to −0.026, reflecting a weakened electrostatic attraction, thereby explaining the increased Gibbs energy of **NiO-U/FOL** compared with **NiO-U/FAL**.

### 3.4. Electronic Structures

Calculations show that the zinc catalyst complexes had a remarkably large HOMO–LUMO energy gap (Figure S7): 1.98 and 2.46 eV for ZnO/FAL and ZnO/FOL, respectively. The uranium doping greatly narrowed the gaps: 0.65 eV for ZnO-U/FAL and 0.86 eV for ZnO-U/FOL. Zinc-based catalysts showed excellent stability when combined with furfural and furfuryl alcohol, thereby avoiding the occurrence of side reactions. Regardless of whether uranium was doped or not, the energy gaps of Cat/FOL were always larger than those of Cat/FAL, indicating that the stability of the former was better than that of the latter. After uranium doping, the HOMO energy level of Cat/FAL and Cat/FOL was destabilized, while the LUMO energy level stabilized, leading to a decrease in the energy gap. This indicates that doping reduced the stability of intermediate complexes. However, despite the stability being weakened, it was still superior compared to the other two metal-based catalysts, and the weakening of the stability was exchanged for an improvement of the catalytic cycle efficiency. When ZnO-U participated in the reaction, the smaller desorption was explainable. Additionally, the calculated energy gaps of Cat/FAL and Cat/FOL for doped and undoped nickel and copper catalysts were very small (less than 0.25 eV). It is known that the GGA PBE functional tends to overestimate HOMO and underestimate LUMO orbital energy. Therefore, hybrid functionals B3LYP and PBE0 were also used to characterize the four systems ZnO/FAL, ZnO/FOL, ZNO-U/FAL, and ZNO-U/FOL. As shown in Figure S11, a more stable HOMO and higher LUMO were obtained using hybrid functionals, consequently giving a wider HOMO–LUMO gap than GGA.

**Figure 5:**
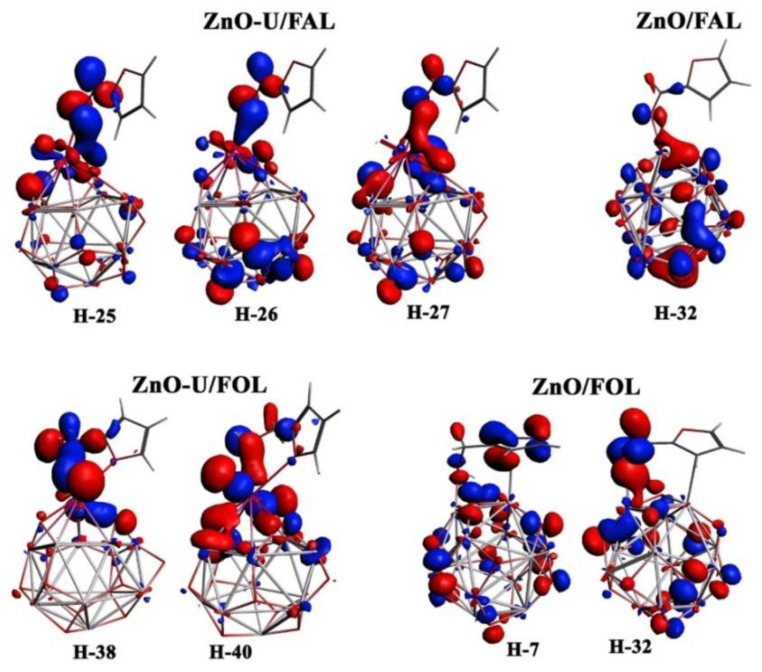
Selected characteristic orbitals of Cat/FAL and Cat/FOL (Cat = ZnO and ZnO-U).

The molecular orbitals confirmed the thermodynamic results that ZnO-U was the most suitable catalyst, according to the analysis of geometric, bonding, and charge properties. In ZnO/FAL and ZnO/FOL, Zn–O1 bonds appeared between the catalyst and organic molecules. They were formed by the donation from O(2p) to Zn(4s). After doping, the U–O bonds in ZnO-U/FAL and ZnO/FOL were the result of overlapping U 5f and O 2p orbitals. Some selected orbitals are illustrated in Figure 5, showing the moderately stable zinc-derived intermediates. In NiO/FAL and NiO/FOL, the overlapping of O/O(2p) and Ni(4ds) was found, as shown in Figure S8. This explains why their formation reactions gave off much more Gibbs free energy. In the doped complexes, U–O1 replaced Ni–O1, featuring the orbital U(5f)–O(2p) overlap. The TM–C bonds in NiO-U/FOL were the same as those in NiO/FOL. Similar changes were found in CuO/FAL and CuO/FOL.

## 4. Conclusions

Two types of catalysts, transition metal oxides (NiO, CuO, and ZnO) and uranium-doped ones (NiO-U, CuO-U, and ZnO-U), were investigated for the catalytic transformation of furfural to furfuryl alcohol by means of relativistic DFT calculations. It was shown that ZnO-U had the best catalytic performance among the six catalysts, only requiring very small reaction free energies of −5.08, −10.54, and 8.12 kJ/mol for the adsorption of furfural, the dihydrogen addition, and the release of furfuryl alcohol, respectively. The catalytic conversion was greatly enhanced by the uranium doping compared with ZnO from a thermodynamic and kinetic perspective. All three TMOs were found to be capable of catalyzing the furfural hydrogenation. However, the uranium doping of NiO and CuO made the whole reaction much harder, whereby either hydrogenation or desorption required overcoming large uphill energy (52.09 or 64.44 kJ/mol). Regarding the intermediates of Cat/FAL and Cat/FOL, furfural/furfuryl alcohol were bound to the catalyst by carbonyl/hydroxyl oxygen, showing the potential reaction selectivity of the catalyst. The calculated bond lengths, bond orders, and charges suggest that uranium doping resulted in much more stable nickel and copper intermediates compared to zinc. Analyses of the electronic structure gave the same results.

## Figures and Tables

**Table 1:** Optimized bond lengths (Å) and bond orders (in parentheses) of Cat/FAL and Cat/FOL complexes.

	M–O1 ^a^	U–O2 ^a^	TM–C	O···H ^a^
NiO/FAL	1.967 (0.49)		2.222 (0.10)	
NiO/FOL	2.208 (0.27)		2.139/2.245 (0.13/0.16)	
CuO/FAL	2.057 (0.41)			
CuO/FOL	2.121 (0.31)			
ZnO/FAL	2.083 (0.36)			1.961 (0.09)
ZnO/FOL	2.201 (0.34)			1.657 (0.18)
NiO-U/FAL	2.382 (0.52)			
NiO-U/FOL	2.571 (0.34)		2.179/2.124 (0.17/0.12)	
CuO-U/FAL	2.366 (0.55)			
CuO-U/FOL	2.607 (0.32)	2.707 (0.17)		
ZnO-U/FAL	2.453 (0.43)			
ZnO-U/FOL	2.611 (0.33)	2.786 (0.15)		

^a^ M denotes the uranium and transition metal atom for uranium-doped and -undoped complexes, respectively. There are three types of oxo atoms. The *endo* oxo atom of the furan ring in furfural and furfuryl alcohol is marked as O2, while the exo oxo one is marked as O1. The oxo atom of the catalyst is not numbered.

## Data Availability

Not applicable.

## Supplementary Materials

Supplementary materials are available online. The following supporting information can be downloaded at https://www.mdpi.com/article/10.3390/molecules27186094/s1: Figure S1. Isomeric structures of NiO/FAL and NiO/FOL; Figure S2. Isomeric structures of NiO-U/FAL and NiO-U/FOL; Figure S3. Isomeric structures of CuO/FAL and CuO/FOL; Figure S4. Isomeric structures of CuO-U/FAL and CuO-U/FOL; Figure S5. Isomeric structures of ZnO/FAL and ZnO/FOL; Figure S6. Isomeric structures of ZnO-U/FAL and ZnO-U/FOL; Figure S7. Energy levels of Cat/FAL and Cat/FOL; Figure S8. Orbital diagrams of NiO/FAL, NiO/FOL, NiO-U/FAL and NiO-U/FOL; Figure S9. Orbital diagrams of CuO/FAL, CuO/FOL, CuO-U/FAL and CuO-U/FOL; Figure S10. Kinetic free energies of zinc-based catalysts adsorbing furfural; Figure S11. HOMO and LUMO energies calculated by functionals PBE, B3LYP and PBE0; Table S1. Relative energies of various oxides isomers; Table S2. Relative energies of various U-doped oxides isomers; Table S3. Calculated reaction energies; Table S4. The sum of the covalent radius and van der Waals radius [41].

## Sample Availability

Samples of the compounds are not available from the authors.
