# Supplementary material for: Uranium-Doped Zinc, Copper, and Nickel Oxides for Enhanced Catalytic Conversion of Furfural to Furfuryl Alcohol: A Relativistic DFT Study
Source: Molecules. 2022 Sep 18;27(18):6094. doi: 10.3390/molecules27186094 (PMC9502827; doi:10.3390/molecules27186094)
Supplement: Supplementary file 1 [file molecules-27-06094-s001.zip › molecules-1910049-supplementary.pdf]

Supporting Information

Uranium-Doped Zinc, Copper, and Nickel Oxides for Enhanced Catalytic Conversion of Furfural to Furfuryl Alcohol: A Relativistic DFT Study

Shuang Li [1,†], Yu-Chang Hou [1,†], Yuan-Ru Guo [2,*] and Qing-Jiang Pan [1,*]

[1] Key Laboratory of Functional Inorganic Material Chemistry (Ministry of Education), School of Chemistry and Materials Science, Heilongjiang University, Harbin 150080, China

[2] Engineering Research Center of Advanced Wooden Materials, Ministry of Education, College of Material Science and Engineering, Northeast Forestry University, Harbin 150040, China

* Correspondence: authors: guoyrnefu@163.com (Y.-R.G.); panqjitc@163.com (Q.-J.P.)

† These authors contributed equally to this work.

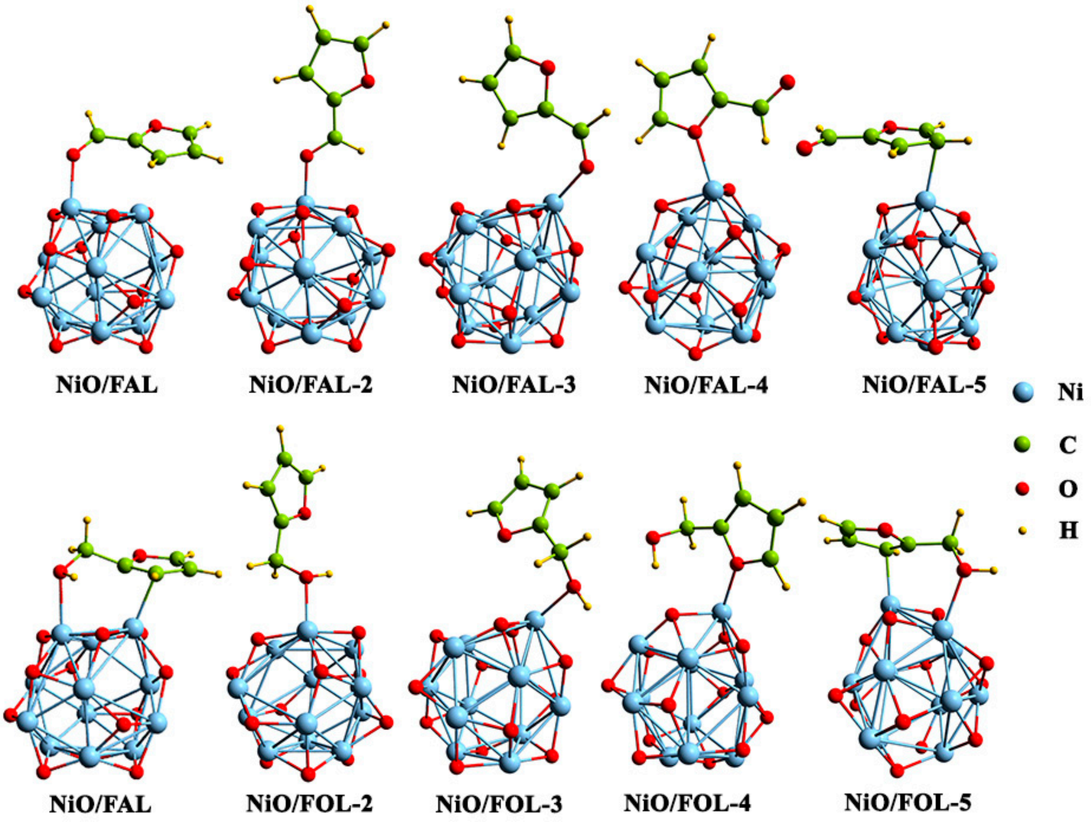

Figure S1. Optimized structures of various isomers NiO/FAL and NiO/FOL.

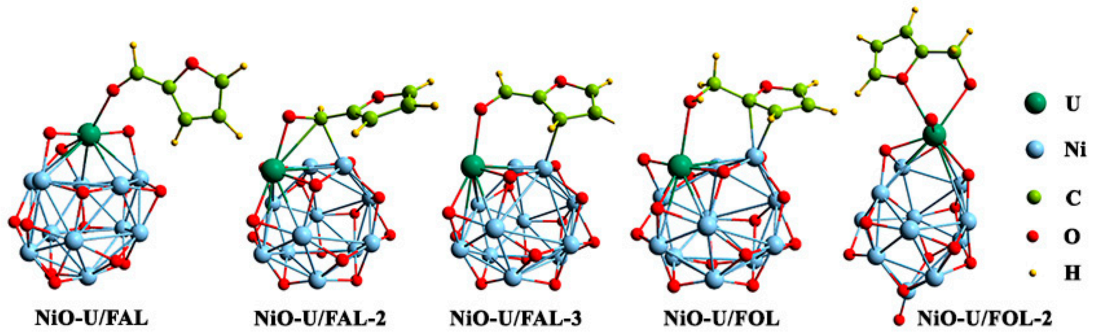

Figure S2. Optimized structures of various isomers NiO-U/FAL and NiO-U/FOL.

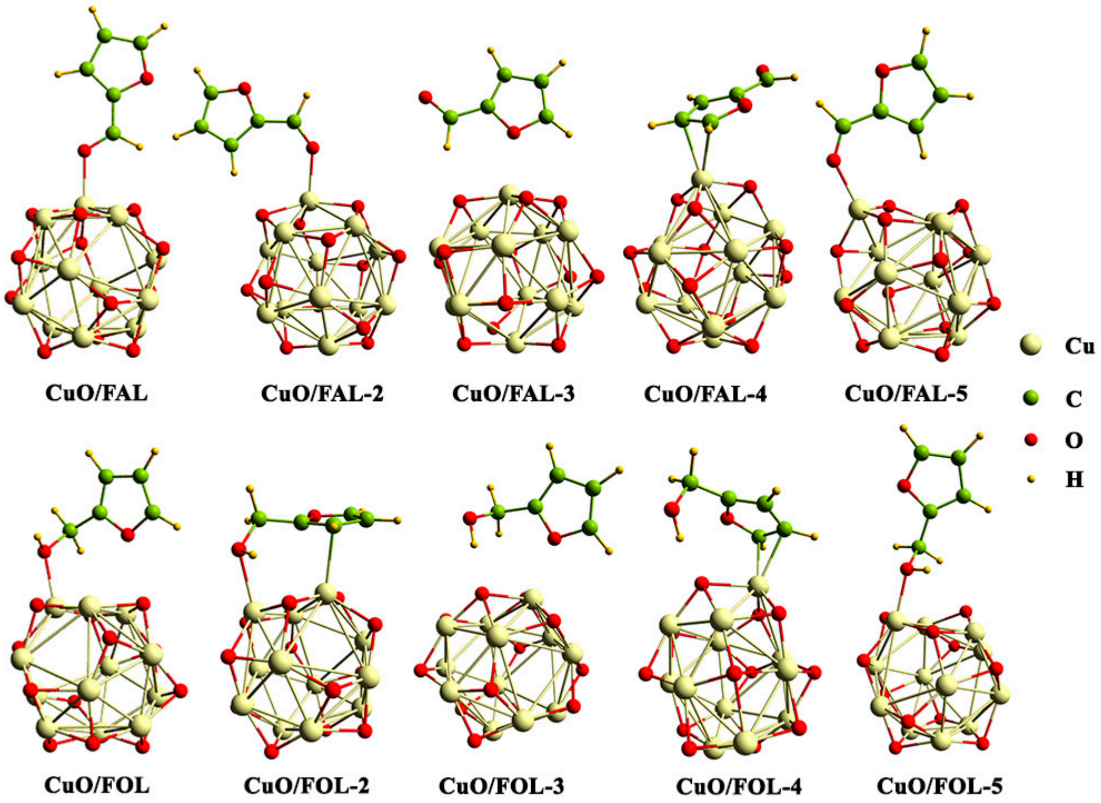

Figure S3. Optimized structures of various isomers CuO/FAL and CuO/FOL.

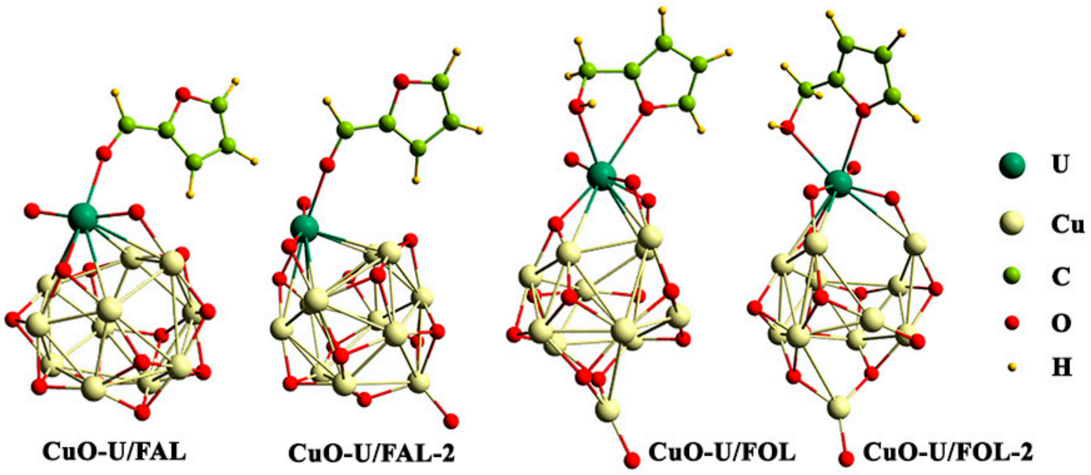

Figure S4. Optimized structures of various isomers CuO-U/FAL and CuO-U/FOL.

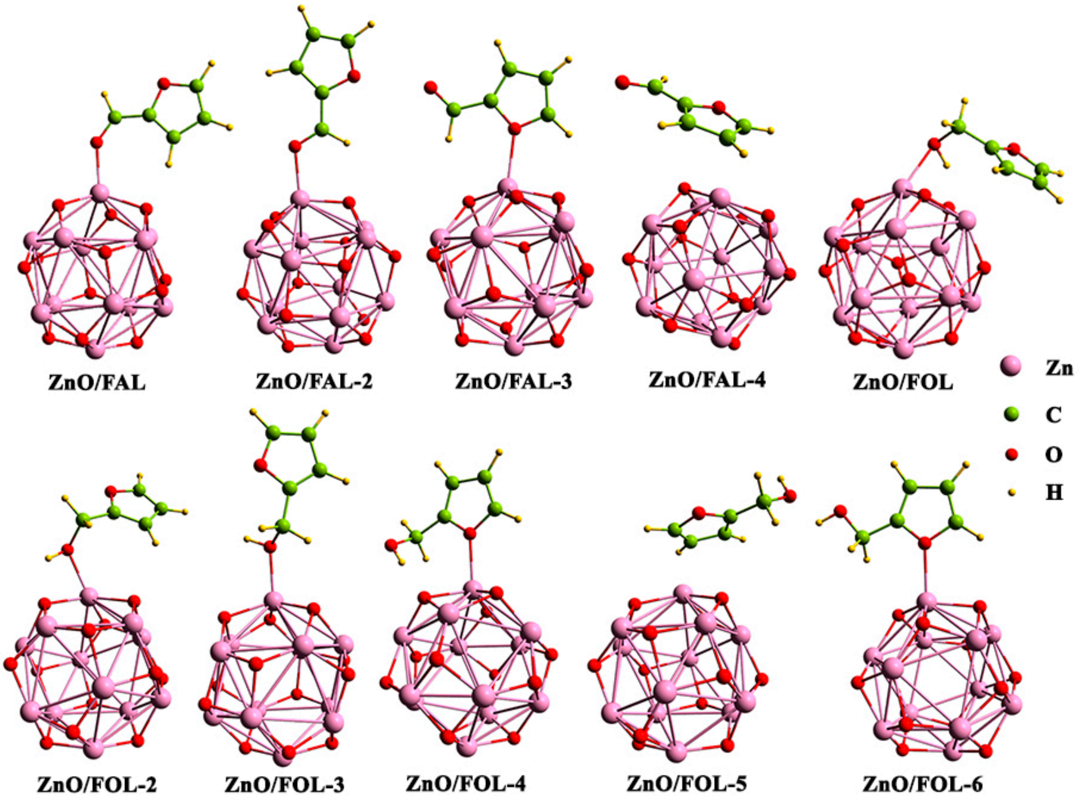

Figure S5. Optimized structures of various isomers ZnO/FAL and ZnO/FOL.

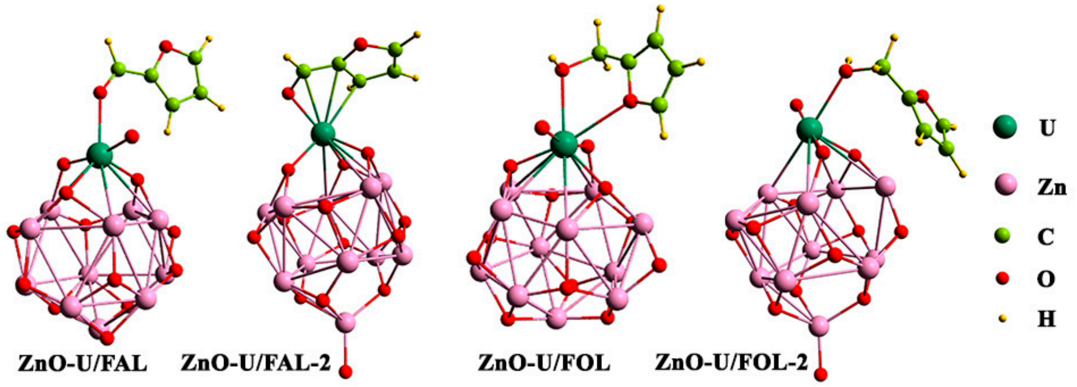

Figure S6. Optimized structures of various isomers ZnO-U/FAL and ZnO-U/FOL.

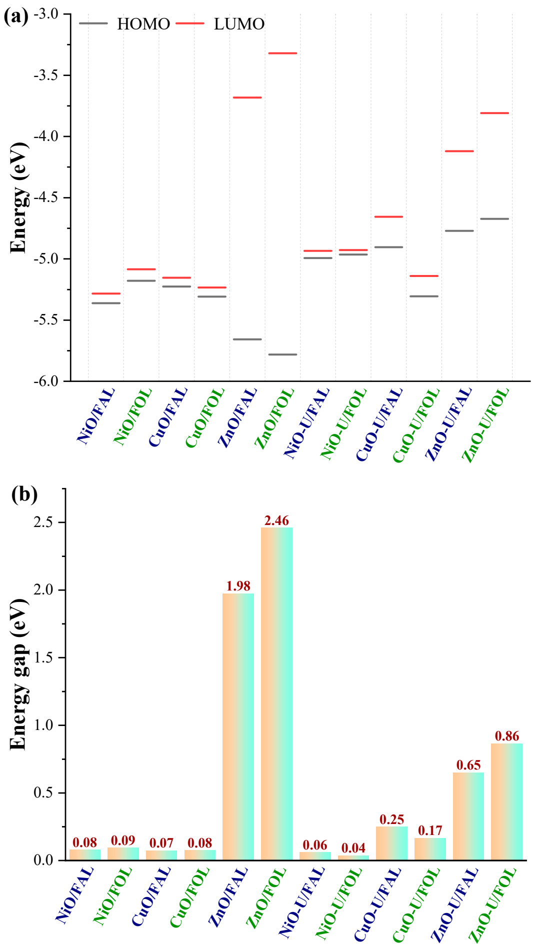

Figure S7. HOMO and LUMO energy levels of Cat/FAL and Cat/FOL (a) and their energy gap values (b).

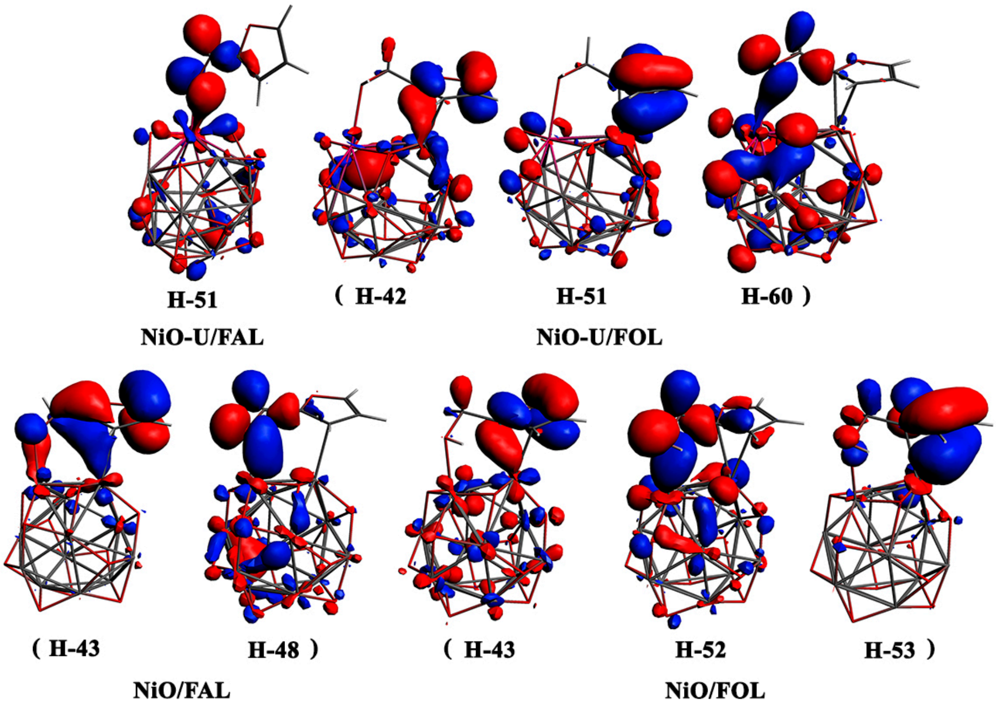

Figure S8. Some orbital diagrams of NiO/FAL, NiO/FOL, NiO-U/FAL and NiO-U/FOL.

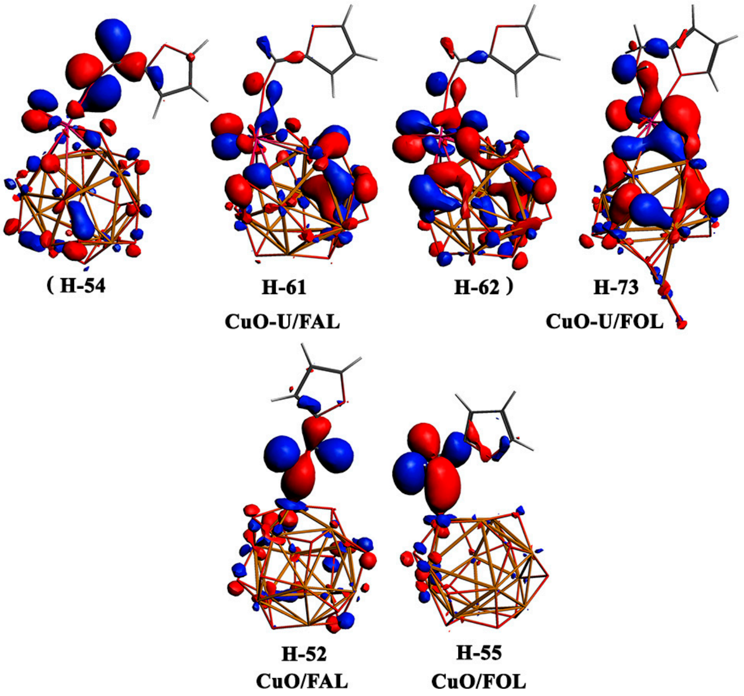

Figure S9. Some orbital diagrams of CuO/FAL, CuO/FOL, CuO-U/FAL and CuO-U/FOL.

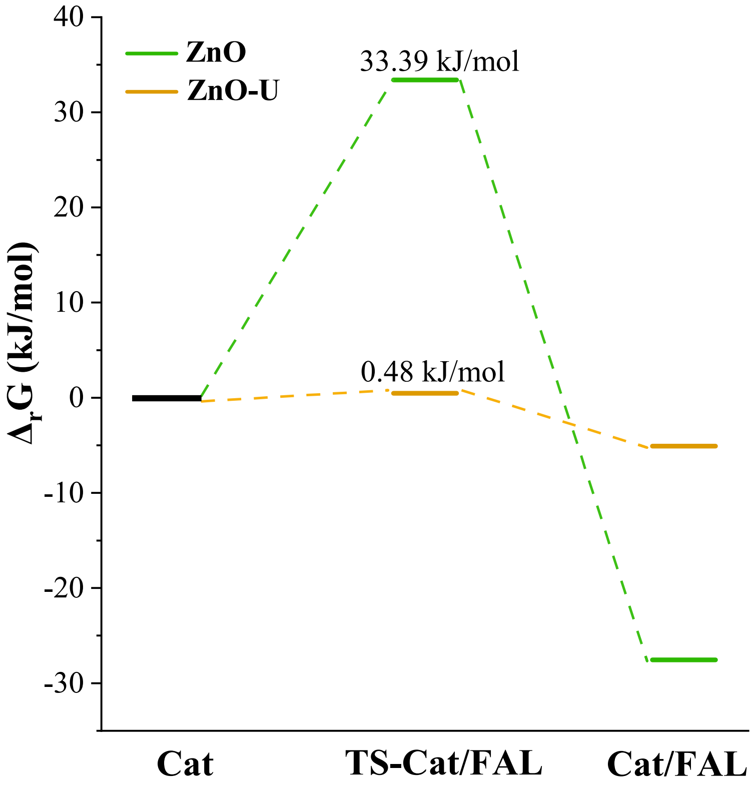

Figure 10. The free energies of zinc-based catalysts adsorbing furfural along the reaction pathway.

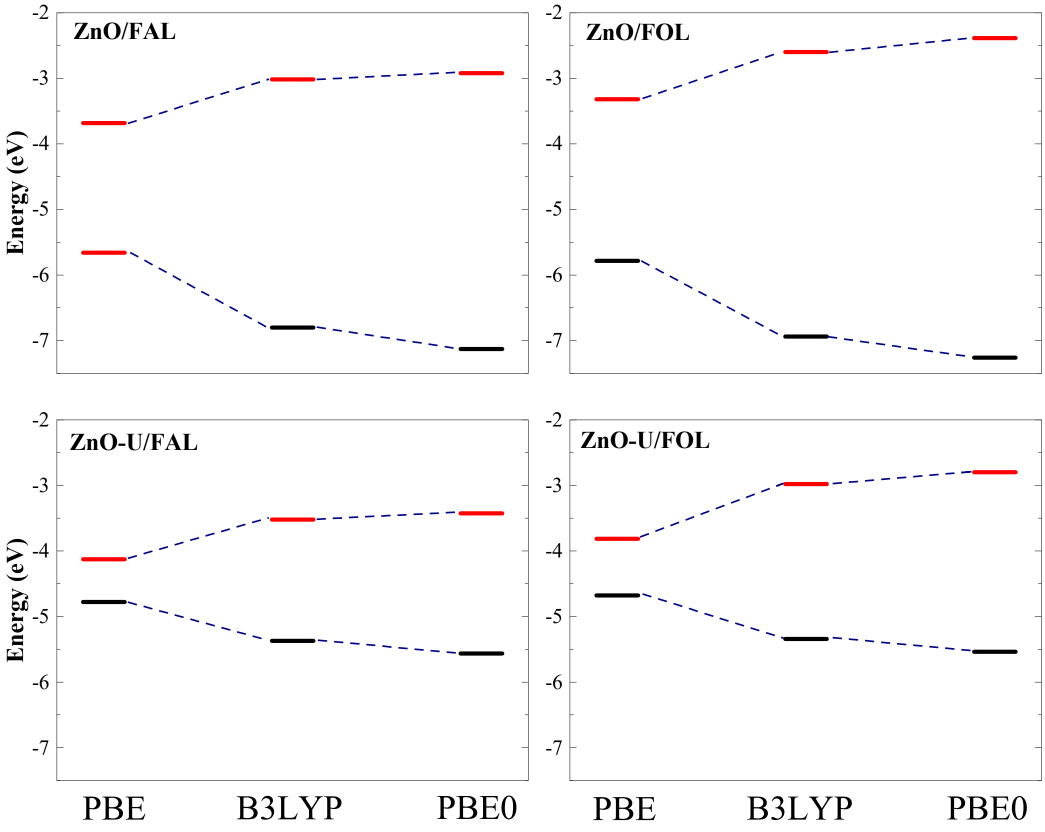

Figure S11. HOMO and LUMO orbitals of ZnO/FAL, ZnO/FOL, ZnO-U/FAL and ZnO-U/FOL calculated by various functionals (PBE, B3LYP and PBE0).

Table S1. Relative energies (kJ/mol) of various isomers Cat/FAL and Cat/FOL (Cat = NiO, CuO and ZnO) compared to the energy-lowest one.

Structures	ΔE	ΔE_0	ΔH	ΔG
NiO/FAL	0.00	0.00	0.00	0.00
NiO/FAL-2	19.50	20.21	21.36	14.40
NiO/FAL-3	43.28	42.55	44.59	34.61
NiO/FAL-4	92.90	88.85	91.98	79.13
NiO/FAL-5	96.75	91.41	94.74	81.58
NiO/FOL	0.00	0.00	0.00	0.00
NiO/FOL-2	38.70	36.69	36.77	31.46
NiO/FOL-3	55.31	53.10	55.59	40.78
NiO/FOL-4	80.68	77.54	80.16	69.39
NiO/FOL-5	66.39	61.68	64.28	58.07
CuO/FAL	0.00	0.00	0.00	0.00
CuO/FAL-2	3.99	3.69	3.68	4.12
CuO/FAL-3	41.34	38.22	40.16	33.38
CuO/FAL-4	42.89	39.79	40.96	36.98
CuO/FAL-5	57.14	52.80	54.01	52.76
CuO/FOL	0.00	0.00	0.00	0.00
CuO/FOL-2	3.68	3.84	3.22	8.35
CuO/FOL-3	36.75	34.67	35.51	30.71
CuO/FOL-4	27.65	25.08	25.31	26.49
CuO/FOL-5	32.29	31.74	32.38	28.58
ZnO/FAL	0.00	0.00	0.00	0.00
ZnO/FAL-2	4.72	4.69	5.06	2.81

ZnO/FAL-3	44.93	42.18	43.59	37.26
ZnO/FAL-4	47.12	44.59	45.93	40.74
ZnO/FOL	0.00	0.00	0.00	0.00
ZnO/FOL-2	13.80	14.05	14.78	7.53
ZnO/FOL-3	15.43	15.13	16.98	0.93
ZnO/FOL-4	22.15	22.25	23.02	16.56
ZnO/FOL-5	47.98	46.52	48.79	37.33
ZnO/FOL-6	56.73	53.28	56.67	39.53

Table S2. Relative energies (kJ/mol) of various isomers Cat/FAL and Cat/FOL (Cat = NiO-U, CuO-U and ZnO-U) compared to the energy-lowest one.

Structures	ΔE	ΔE_0	ΔH	ΔG
NiO-U/FAL	0.00	0.00	0.00	0.00
NiO-U/FAL-2	21.57	22.20	18.93	34.42
NiO-U/FAL-3	53.24	52.43	50.42	62.42
NiO-U/FOL	0.00	0.00	0.00	0.00
NiO-U/FOL-2	66.87	62.53	68.78	41.99
CuO-U/FAL	0.00	0.00	0.00	0.00
CuO-U/FAL-2	207.54	201.17	205.80	190.56
CuO-U/FOL	0.00	0.00	0.00	0.00
CuO-U/FOL-2	14.67	14.96	14.86	15.53
ZnO-U/FAL	0.00	0.00	0.00	0.00
ZnO-U/FAL-2	434.97	426.82	427.67	425.37
ZnO-U/FOL	0.00	0.00	0.00	0.00
ZnO-U/FOL-2	533.54	527.75	532.04	515.60

Table S3. The calculated reaction energies (kJ/mol) for adsorption, hydrogenation and desorption triggered by catalysts.

Reactions		$\Delta_r E$	$\Delta_r E_0$	$\Delta_r H$	$\Delta_r G$
Step I : Adsorption	NiO + FAL = NiO/FAL	-92.68	-89.93	-89.31	-33.93
Step II: Hydrogenation	NiO/FAL + H_2 = NiO/FOL	-87.04	-25.24	-59.05	-18.56
Step III: Desorption	NiO/FOL = NiO + FOL	108.81	104.85	105.29	45.00
Step I : Adsorption	CuO + FAL = CuO/FAL	-74.28	-70.33	-68.09	-24.13
Step II: Hydrogenation	CuO/FAL + H_2 = CuO/FOL	-79.06	-19.31	-52.48	-13.06
Step III: Desorption	CuO/FOL = CuO + FOL	82.44	79.32	77.51	29.70
Step I : Adsorption	ZnO + FAL = ZnO/FAL	-78.64	-74.87	-72.76	-27.53
Step II: Hydrogenation	ZnO/FAL + H_2 = ZnO/FOL	-83.09	-23.56	-57.54	-13.50
Step III: Desorption	ZnO/FOL = ZnO + FOL	90.82	88.12	87.24	33.53
Step I : Adsorption	NiO-U + FAL = NiO-U/FAL	-130.83	-127.10	-125.18	-79.00
Step II: Hydrogenation	NiO-U/FAL + H_2 = NiO-U/FOL	-21.36	37.65	3.31	52.09
Step III: Desorption	NiO-U/FOL = NiO-U + FOL	81.29	79.13	78.81	19.41
Step I : Adsorption	CuO-U + FAL = CuO-U/FAL	-135.80	-131.42	-129.99	-80.61
Step II: Hydrogenation	CuO-U/FAL + H_2 = CuO-U/FOL	-37.30	19.05	-9.78	8.65
Step III: Desorption	CuO-U/FOL = CuO-U + FOL	102.19	102.05	96.71	64.47
Step I : Adsorption	ZnO-U + FAL = ZnO-U/FAL	-55.46	-52.71	-49.97	-5.08
Step II: Hydrogenation	ZnO-U/FAL + H_2 = ZnO-U/FOL	-78.29	-17.81	-51.29	-10.54
Step III: Desorption	ZnO-U/FOL = ZnO-U + FOL	62.85	60.21	58.20	8.12

Table S4. The sum of the covalent radius and van der Waals radius (Å) of Ni, Cu, Zn, U, H atoms with O, C atoms [1].

Covalent radius					
Bond atoms	Ni	Cu	Zn	U	H
O	1.90	1.98	1.88	2.62	0.97
C	2.00				
Van der Waals radius					
Bond atoms	Ni	Cu	Zn	U	H
O	3.15	2.92	2.91	3.92	2.72
C	3.33				

Reference

1. Kim, S.; Chen, J.; Cheng, T.; Gindulyte, A.; He, J.; He, S.; Li, Q.; Shoemaker, B. A.; Thiessen, P. A.; Yu, B.; Zaslavsky, L.; Zhang, J.; Bolton, E. E., PubChem in 2021: new data content and improved web interfaces. *Nucleic Acids Research* **2021,** 49, (D1), D1388-D1395.
